# Comparison of quit rates among exclusive cigarette smokers at Tobacco Control Center in Qatar: telephone versus face-to-face consultation

**DOI:** 10.1186/s12875-025-02858-2

**Published:** 2025-05-15

**Authors:** Muslem M. Aljaafar, Silva Kouyoumjian, Gafar Mahmoud, Ahmad AlMulla

**Affiliations:** Tobacco Control Center, WHO Collaborating Center, Department of Medicine, Hamad Medical Corporation, P.O. Box 3050, Doha, Qatar

**Keywords:** Smoking, Cessation, Telephone counseling, Abstinence, Qatar

## Abstract

**Background:**

During the coronavirus 2019 pandemic, smoking cessation services were delivered by telephone instead of routine face-to-face care. The objective of this study was to (1) determine if telephone care increases smoking quit rate compared to face-to-face intervention and (2) investigate factors associated with successful smoking cessation.

**Methods:**

A retrospective study design was utilized. Random selection of patients from two groups (face-to-face care and telephone care) that completed 3 follow-up sessions in Tobacco Control Center in Hamad Medical Corporation in Qatar was conducted from April 2020 to September 2021. Quit rates were compared at 4-week, 8-week and 12-week follow-up visits and a multivariable logistic regression analysis was conducted to obtain factors related to successful quitting at 12-week follow-up.

**Results:**

A total of 138 patients were included in this study (69 patients for each group). At 12-week follow-up, 31.9% of patients of telephone care (*n* = 22) succeeded in quitting smoking, while only 20.3% (*n* = 14) remained abstinent in the face-to-face care group. Telephone care compared with standard care increased the odds of success in stopping smoking (AOR = 3.279; 95% Cl: 1.191–9.026). Also, smokers with a previous quit attempt were significantly related to stopping smoking successfully (AOR = 4.724; 95% Cl: 1.131–19.727). Higher consumption of self-reported cigarette smoking was statistically associated with lower success rates in smoking cessation (AOR = 0.919; 95% Cl: 0.874–0.966).

**Conclusion:**

Our data suggests that providing telephone care seems more effective in smoking cessation treatment compared with routine face-to-face intervention. However, further formal assessment as randomized clinical trial needs to be conducted for more evaluation.

**Supplementary Information:**

The online version contains supplementary material available at 10.1186/s12875-025-02858-2.

## Introduction

Tobacco use is the leading preventable cause of death worldwide and a modifiable risk factor for non-communicable diseases (NCDs) [1]. Qatar is among the countries implementing national strategies and population programs that focus on promoting healthy behaviors and lifestyles [2]. Given the changes in modern lifestyle and the increased likelihood of developing a chronic disease, one of the national strategy’s focus is to combat smoking and reduce tobacco consumption [2, 3]. According to a recent population-based study, the prevalence of tobacco smoking in Qatar has dropped by 15.2% between 2000 and 2019 (from 36.7 to 21.5%) [4].

Qatar promotes smoke free society and environment since 2002 and implements more comprehensive measures every year such as the updated law no. 10 of 2016 on the Control of Tobacco and its Derivatives that ensure restrictions for tobacco-related businesses, reduces the demand through price increases and provides accessible tobacco cessation services with support in treatment cost among others [5–7]. Recently in 2021, Hamad Medical Corporation’s (HMC) Tobacco Control Center (TCC) was re-designated by the WHO as a collaborating center for treating tobacco dependence, knowing that it is currently the only kind in Qatar and in the Gulf Cooperation Council region. TCC has aligned its goals with Qatar National Vision of 2030 to steadily work towards further reduction in the prevalence of tobacco, since treating tobacco dependence and assisting cessation is one of the national priorities. The center also conducts scientific research, promotes health awareness, and trains clinicians and specialists about nicotine dependence treatment with the collaboration of WHO [5].

Tobacco cessation at any instant is a significant health gain at the individual and the public level resulting in reduced societal costs and health care burden. According to Article 14 of the Framework Convention on Tobacco Control assisting tobacco cessation is an important component of tobacco control and one of the most cost-effective intervention [8]. The success of tobacco cessation can be enormous and can have a substantial impact on smoking rates, prevention of tobacco related diseases and enhancement of public health [9]. The probability of quitting smoking on any quit effort is low. According to the Australian Cancer Council it takes 12–14 attempts before a smoker successfully can quit [10]. Furthermore, a recent study estimated that a current smoker tries to quit on average 30 times or more before successfully quitting for 1 year or longer [11]. Smokers have a success rate for unassisted quit attempts of only 3–5% [12], however cessation programs show smoking abstinence rates of up to 38% [13]. Based on data pooled from randomized trials of smoking cessation, shows that a minimal intervention of 1 min (brief advice) would increase the rate of quitting by relative risk of 1.66, 95% confidence interval 1.42–1.9 when compared with no intervention [14]. It is well established that evidence-based treatments for tobacco use and dependence such as behavioral counseling and pharmacotherapy, can increase cessation success [15, 16].

Tobacco cessation services in Qatar are mainly provided by TCC’s clinics embedded within HMC and through clinics in the Primary Health Care Corporation [17]. The center adopts a well-established effective approach of therapy where cessation counseling is combined with medication [5]. During the COVID-19 pandemic as a safety measure TCC similar to other outpatient clinic services in HMC was utilizing telephone consultations when physical attendance was not allowable [18]. The integration of technology into behavioral health education and therapy has been established across the globe [19, 20]. Telehealth have greatly expanded to provide medical and preventive care for populations worldwide during and after the coronavirus disease 2019 pandemic [21]. The available evidence from a recent review shows that telephone counselling conducted in dental clinics can be more effective than usual care for tobacco cessation [22]. Also, according to a randomized controlled noninferiority trial the efficacy of the internet-based video counseling program for smoking cessation was not inferior to that of the standard face-to-face clinic visit program [23]. Telephone counseling provides convenient and immediate access to services and accelerated progress toward smoking cessation compared to smokers who need to visit or contact the healthcare center to initiate treatment [24, 25].

In Qatar, phone-based counseling program demonstrated acceptability and promising efficacy as found in the literature [26]. However, a recent study found that smoking cessation counseling provided by telephone was less effective mode of treatment when compared to traditional face-to-face encounters [17]. Tobacco treatment modalities vary greatly and more recently are evolving; therefore, it is essential to investigate the impact of various treatment methods for tobacco dependence and its efficacy. Furthermore, studies have been conducted to determine the predictors associated with successful smoking cessation such as older age, marriage, higher income, better mental health status, low level of nicotine dependence, and home rules against smoking [27, 28]. Knowing that there is limited published evidence on tobacco cessation in Qatar and a lack of knowledge about smoking cessation success, it is also imperative to study about the factors affecting the success of quitting. All this would enable us to improve effectiveness and expansion implementation of treatment methods to have a greater chance of successful cessation. Hence, our study used a retrospective approach to (1) assess the effectiveness of Tobacco Control Center’s two cessation methods by comparing the smoking quit rate among telephone care group versus face-to-face care group (2) identify the determinants that influence successful cessation of cigarette smoking. The goal of this study is to compare TCC’s two cessation treatment methods and determine which led to the highest smoking quit rate. Major gains can be attained through better knowledge. Findings will provide directions for future research studies, and aid decision-making in tobacco cessation health programs and policies.

## Methods

### Study design, setting, and participants

This study used a retrospective, cross-sectional study design. This study was conducted in Tobacco Control Center in Hamad Medical Corporation in Qatar. TCC is a WHO Collaborating Center, this commitment entitles the Center to carry out a series of activities in support of the WHO’s mandated international health agenda in efforts of tobacco control and prevention in the country. HMC is a large Joint Commission International-accredited group of nine tertiary hospitals across different regions in Qatar affiliated with the government. TCC has established five dedicated clinics across HMC hospitals (Medical city, Hamad General Hospital, Al Wakra Hospital, Al Khor Hospital and Hazm Mebaireek General Hospital) where they operate in a uniform manner. Participants were selected from the individuals registered in smoking cessation clinics only at the Tobacco Control Center in the Medical city from April 2020 to September 2021. During this period, tobacco cessation service was intermittent either face-to-face or through telephone consultations due to the COVID-19 pandemic. Our target population were exclusive cigarette smokers who were aged 18 years or older who used the smoking cessation program (1st consultation session with 3 follow-up sessions) either in person or via telephone. TCC adopts a multidisciplinary approach with systematic counseling offering pharmacotherapy treatment such as the nicotine replacement therapy (NRT), varenicline and bupropion. Within the program smoking cessation encounters are usually twenty minutes in duration for the initial visit and ten minutes for each follow-up encounter. TCC aims to offer individualized consultations and regular follow-up visits (after 2–4 weeks) based on long-term support. Throughout the program, the physician counsels and prescribes the medical treatment and deals with relapses, the psychologist provides behavioral support and problem-solving skills, and the patient educator enhances patient’s motivation as well as the nurse. Also, a non-pharmacological smoking cessation program such as laser therapy is offered to help people including the youth to stop smoking. The telephone care group received similar treatment program services involving TCC professionals during the 1st consultation and follow-up visits, however over the phone with medications either mailed by a courier to their home address or offered the option for pick-up from the pharmacy. The norm in TCC is that consultations whether face-to-face or via telephone are conducted by the same smoking cessation specialist unless the patient has other preferences or in case of unforeseen circumstances.

### Sample size and sampling

Studies of behavioral interventions for smoking cessation suggest a range of cessation rates with an average of 20% after 1 year [29–32]. With a two-sided alpha of 7% and 95% power, a minimum sample size of 125 participants was required. Allowing for a 10% missing data dropout, a total of 138 patients was needed for recruitment with 69 patients in each group. Participants enrolled in TCC smoking cessation program whether face-to-face or via telephone were selected if they had completed the 3 follow-up sessions. The telephone care group of participants was randomly selected from a list of all cigarette smokers who completed 1st telephone consultation with 3 telephone follow-up consultations between April 2020 and September 2021 during the Coronavirus pandemic, while the face-to-face care group was randomly selected from a list of those who attended 1st consultation physically at TCC in Medical City with 3 in person follow-ups visits during the same period. From patient lists, every other patient fulfilling the above criteria was selected until the desired sample size was reached.

### Ethical review

Ethical approval was granted by the institutional review board in Hamad Medical Corporation in Doha, Qatar (MRC-01-22-398). Patient consent was not required in this study because it was a retrospective study without any patient interaction. Furthermore, the patient during the first consultation visit, when filling out the assessment form in TCC, is asked if he consents his file to be used for scientific research purposes with no identifiers that link to his name, age, or to any personal data.

### Data collection

The data collection tool for both groups was TCC’s patient assessment form a comprehensive questionnaire developed by an extensive literature review with consultation of experts in the field of tobacco cessation found in S1 Appendix in Supplementary Information (SI) file. It is a multicomponent questionnaire designed to capture all relevant demographic and clinical data including age, gender, nationality, marital status, education, and existing comorbidities. In relation to smoking, the amount and duration of cigarette smoking, the Fagerström Test for Nicotine Dependence score [33], having smokers at the living place, the importance and chances of quitting, presence, number and success of previous quitting attempt, and mode of cessation treatment delivered (face-to-face or telephone) were collected. Data was extracted and entered during November 2022– March 2023.

### Data analysis

Extracted data was coded and entered into the Statistical Package for Social Sciences (IBM SPSS statistics; version 29; Armonk, NY: IBM Corporation program). 50% of data entry was reviewed and repeated by a different person to verify and validate the accuracy of the process. Descriptive analysis was performed for the characteristics of the participants and data were presented as frequencies and percentages for categorical variables and mean ± standard deviation (S.D.) for continuous variables. The primary outcome for the two modes of cessation treatment delivered was cigarette smoking quit rate at 1st follow-up which is defined as self-reported abstinence for the 14 days after the first consultation visit whether it was in-person or via telephone, however depending on the patient availability it can range between 2 and 4 weeks. The 2nd follow-up is defined as self-reported abstinence for a month after the 1st follow-up ranging between 6 and 8 weeks. The 3rd follow-up is defined as self-reported abstinence for a month after 2nd follow-up ranging between 10-12-weeks. However, for better comprehension throughout the article we will refer to it as a 4-week, 8-week and 12-week follow-up quit rate. It was defined as the number of smokers who received tobacco services and successfully achieved quitting during each of these follow-up visits. Chi-square testing and Chi-square for trend (categorical ordinal variables) was used to examine contrasts in quit rates by in-person method and telephone counseling type. Comparisons of quit rates between independent groups (e.g., nationality and other demographic variables) were conducted and t-tests were used to compare means between independent groups. A univariate binary logistic regression test was used to assess the relationship between quit rate at different follow-up visits and other covariates. Multivariate binary logistic regression test was then used to incorporate covariates with statistical significance into the models. Entry of variables derived from the univariate analysis into the model was less restrictive (*p* < 0.25) than for the multivariate regression model (*p* < 0.05), because its purpose is to identify potential predictor variables rather than to test a hypothesis.

## Results

### Sample characteristics by treatment method

A total of 138 exclusive smokers were sampled for the study (69 in each telephone and face-to-face care group). Sample included almost all males. The mean age of those who received telephone consultation was 39.5 ± 9.4 years, 87.0% were non-Qataris, 65.2% had a university education, 75.4% were married and 33.3% had health comorbidities. For the face-to-face consultation group the mean age was 40.4 ± 10.3, 94.2% were non-Qataris, 56.5% had a university education, 75.4% were married and 46.4% had comorbidities. None of the baseline socio-demographic variables differed between the two groups (Table 1).

**Table 1:** Sample characteristics by treatment method

	Telephone	Face-to-Face	*p*-Value
	*n* (%) = 69	*n* (%) = 69	
**Demographic characteristics**			
**Gender** (%)			
Male	68 (98.6%)	69 (100%)	0.316
Female	1 (1.4%)	0	
**Age**, mean (SD)	39.5 (± 9.4)	40.4 (± 10.3)	0.334
**Nationality** (%)			
Qatari	9 (13.0%)	4 (5.8%)	0.145
Non-Qatari	60 (87.0%)	65 (94.2%)	
**Education status** (%)			
Secondary or less	24 (34.8%)	30 (43.5%)	0.295
University	45 (65.2%)	39 (56.5%)	
**Marital status** (%)			
Single/Divorced	17 (24.6%)	17 (24.6%)	1.000
Married	52 (75.4%)	52 (75.4%)	
Health problems* (%)			
Yes	23 (33.3%)	32 (46.4%)	0.118
No	46 (66.7%)	37 (53.6%)	
**Tobacco use**			
Number of years of cig. Smoking (SD)	18.1 (± 10.1)	17.0 (± 10.4)	0.737
Number of cig. Smoked per day (SD)	26.2 (± 11.7)	25.0 (± 12.3)	0.729
**Number of cig. Smoked per day**			
0–10	5 (7.2%)	14 (20.3%)	0.558
11–20	31 (44.9%)	16 (23.2%)	
21–30	13 (18.8%)	23 (33.3%)	
> 30	20 (29.0%)	16 (23.2%)	
**Quitting**			
**Importance of quitting**			
Very important to quit now	47 (68.1%)	60 (89.6%)	0.009
Important to quit now	20 (29.0%)	6 (9.0%)	
Neutral	2 (2.9%)	1 (1.5%)	
**Chances of quitting success**			
Excellent (80% and above)	31 (48.4%)	26 (38.8%)	0.078
Very high (60–79%)	22 (34.4%)	21 (31.3%)	
High (50–59%)	8 (12.5%)	12 (17.9%)	
Low (less than50%)	3 (4.7%)	8 (11.9%)	
**Past quit attempts**			
Attempt to quit before (%)			
Yes	51 (75.0%)	51 (73.9%)	0.884
No	17 (25.0%)	18 (26.1%)	
Number of past quit attempts			
1–5 times	35 (94.6%)	40 (95.2%)	0.896
> 5 times	2 (5.4%)	2 (4.8%)	
Successful quit attempts (%)			
At least one month or more	17 (45.9%)	20 (60.6%)	0.220
Less than one month	20 (54.1%)	13 (39.4%)	
**Exposure to secondhand smoke**			
Number of adult household members who smoke	1.08 (± 0.3)	1.18 (± 0.4)	0.054
Fagerstrom test for Nicotine Dependence			
Very low (0–2)	5 (7.2%)	6 (8.7%)	1.000
Low (3–4)	8 (11.6%)	4 (5.8%)	
Average (5)	9 (13.0%)	12 (17.4%)	
High (6–7)	20 (29.0%)	22 (31.9%)	
Very high (8–10)	27 (39.1%)	25 (36.2%)	

*High blood pressure, diabetes, asthma, chronic obstructive pulmonary disease, epilepsy, cancer, cardiovascular, high cholesterol, obesity, chronic kidney, mental health and others

Data are shown as number (%) or mean (standard deviation)

The mean duration of cigarette smoking was 18.1 ± 10.1 in the telephone care group, while it was 17.0 ± 10.4 in the face-to-face care group. The average number of cigarettes smoked per day was 26.2 ± 11.7 and 24.9 ± 12.3 in the telephone and face-to-face care groups, respectively. 44.9% smoked 11–20 cigarettes per day in the telephone care group, while 33.3% smoked 21–30 cigarettes in the face-to-face care group. However, both groups had a very high score [8–10] in the Fagerstrom test for Nicotine dependence though not statistically significant. The only significant factor between the two groups was the importance of quitting. The majority of face-to-face care group (89.6%) considered it very important to quit and 68.1% of telephone care patients considered it important (*p* = 0.009) (Table 1).

### Quit rate by treatment method

**Fig. 1:**
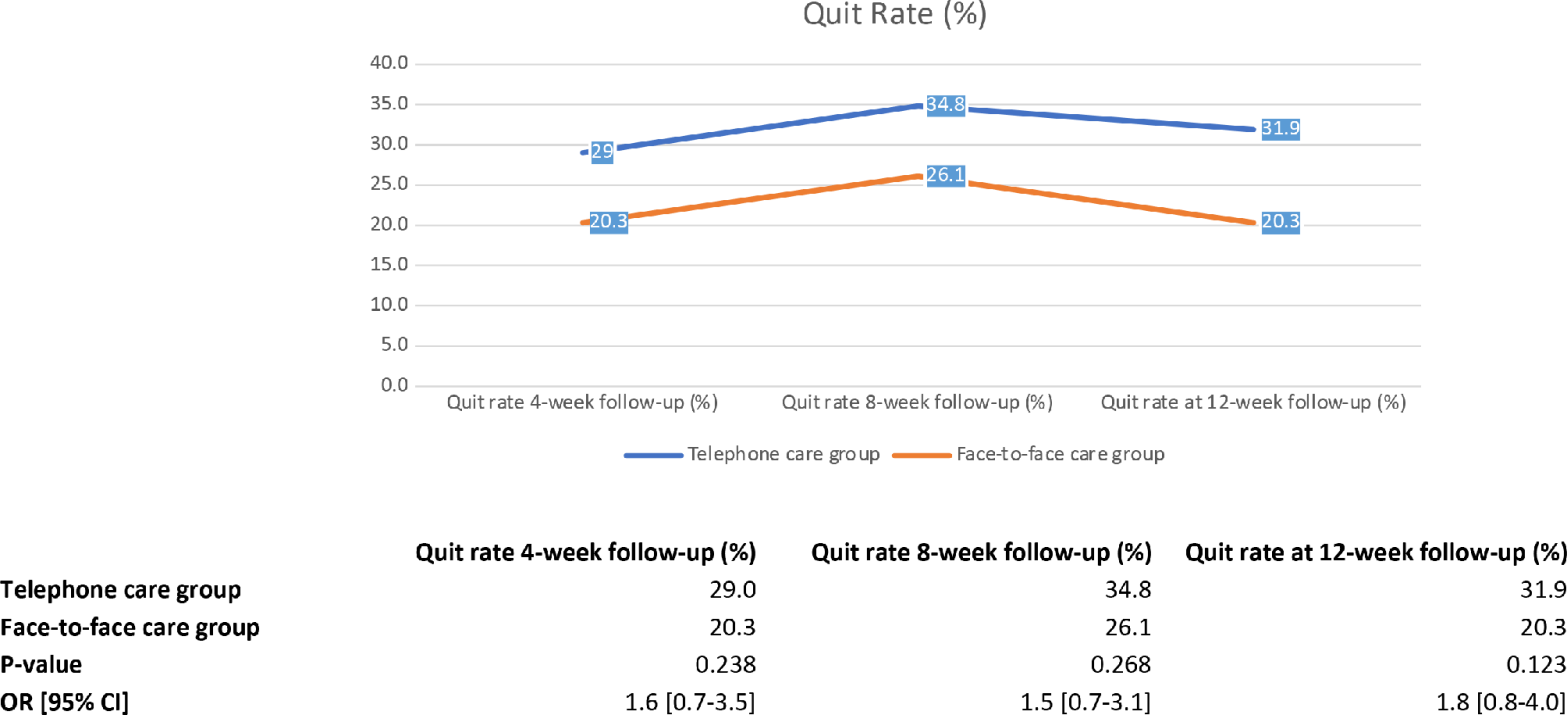
Quit rate between telephone consultation and face-to-face consultations at 4-week, 8-week, 12-week follow-up visits

Figure 1 shows that the quitting rate of the smokers from both groups rose slightly between 4-week and 8-week follow-up visit. The highest quit rate was at the 8-week follow-up, 34.8% (*n* = 24) and 26.1% (*n* = 18) of participants in the telephone and face-to-face consultation groups, respectively. Also, 31.9% (*n* = 22) and 20.3% (*n* = 14), respectively, had achieved self-reported abstinence at 12-week. The quitting rate for the telephone care group remained higher than those in the face-to-face group throughout the period. Neither of these comparisons was statistically significant (Fig. 1).

### Predictors of cessation at the 12-week follow-up visits

Logistic regression was carried out to assess the effects of several independent variables on the quit rate of the participants at the 12-week follow-up. The results of the multivariate binary logistic regression models are presented in Table 2. The logistic regression model for cessation was statistically significant (p-value < 0.001) compared to the null model with a chi-squared value (χ2) of 31.35. For every increase in the number of cigarettes smoked per day by the smokers, the odds of successful quitting were decreased by (Adjusted odds ratio (AOR) = 0.919, 95% CI 0.874–0.966). Those who had tried to quit from before were more likely to quit smoking at the 12-week follow-up AOR = 4.724, 95% CI 1.131–19.727, respectively. Those who received telephone counseling were more likely to quit at the 12-week follow-up, AOR = 3.279, 95% 1.191–9.026.

**Table 2:** Multivariate binary logistic regression of the determinants of quitting smoking among patients attending TCC at 12-week follow-up visit

	Adjusted odds ratio (AOR) (95% CI)	*p*-value
**Demographic characteristics**		
Age	0.943 (0.861–1.032)	0.203
Education		
Secondary or less	1.0 (ref.)	
University	0.388 (0.144–1.045)	0.061
Marital status		
Single	-	-
Married	-	-
**Tobacco dependence**		
Number of cig/days	**0.919 (0.874–0.966)**	**0.001**
Number of years of smoking	1.040 (0.955–1.133)	0.365
**Quitting**		
Tried to quit in the last year		
No	1.0 (ref)	
Yes	**4.724 (1.131–19.727)**	**0.033**
Chances in quitting smoking		
Low (≤ 59%)	1.0 Ref	
High (≥ 60%)	0.941(0.341–2.596)	0.906
Importance of quitting		
Important	1.0 Ref	
Very Important	3.189(0.734–13.860)	0.122
**Mode of cessation treatment delivery**		
Face-to-Face	1.0 Ref	
Telephone	**3.279 (1.191–9.026)**	**0.022**

## Discussion

Evidence from the literature shows that telephone counseling in smoking cessation services increases chances of quitting [25, 34, 35]. Multiple studies assessed its effectiveness and level of interest in the context of the COVID-19 pandemic since it can be feasibly applied [18, 21, 26, 36]. Participants in general expressed a strong interest in accepting the rapid transition from in-person to telephone-based consultation [18, 26]. Our study compared the success rates of quitting by evaluating the findings from smokers receiving telephone consultations versus smokers who underwent the treatment service in face-to-face encounters at TCC clinics and examined the predictors associated with quitting.

We found that the quit rate was higher among cigarette smokers who received the telephone service than who physically visited the clinics at TCC though this was not significant. The necessity of physically visiting the clinic may be one of the reasons for the lower success rate in the face-to-face group particularly during the pandemic. Telephone counseling provides easier access and prevents participants from missing their regular counseling leading to better cessation success rate. Providing medical care to patients remotely is considered useful as it minimizes waste of time visiting the healthcare institution and reduces waiting time for physician consultations [23].

In our study, the quit rate on 12-week follow-up was 31.9% for the telephone group and 20.3% for the face-to-face group (p-value = 0.123). Based on evidence, quit rates range from 2 to 50% with an average of 15.2% among participants receiving pharmacotherapy and behavioral support versus minimal intervention [37]. Our successful quitting rates on 12-week is comparable to former studies of TCC conducted over the years reporting a high range between 35 and 40% (personal communication). Unfortunately, data about the quitting rates among smokers in the general population of Qatar are very limited or not published. For example, among a total of 314 smokers in Qatar that were randomized into two groups: intervention (*n* = 167) and control (*n* = 147), smoking cessation rates were higher in the intervention group at 12 months (23.9% vs. 16.9%) [38]. The much higher quit rates in our study could be attributed to the ongoing concern about the COVID-19 pandemic when smokers were more motivated to quit and were more open to smoking consultations for smoking cessation.

There are multiple studies comparing phone-based interventions to traditional visit-based counseling [23, 39, 40] and research on smoking cessation suggests that they can increase tobacco quitting by 25–50% [34]. Counseling over the phone is convenient with fewer logistical barriers and increased service uptake [41]. Tech-driven approaches deserve attention which could be combined with face-to-face approach improving the rate of smoking cessation [42]. Based on estimates from clinical trials, providing behavioral support either face-to-face or by telephone for people using pharmacotherapy to quit smoking is likely to increase the chance of success by about 10–20% [43]. Telephone consultation combines both pharmacotherapy and behavioral therapy and has been shown to have fewer hinders typically associated with lower attendance and fewer other costs associated with quitting [41].

Research investigating the numbers of telephone counselling calls and different timings of call-backs would be recommended, as there is some evidence that suggests that higher numbers of calls may be more effective than a single call [25]. Another review suggests that telephone counseling has a pragmatic effect on interactional aspects of psychological therapy [22]. There are concerns about the delivery of counseling therapy over the phone, however according to a systematic review there is no support for the idea that telephone has a detrimental effect on interactional aspects of psychological therapy [44]. Also, given the conservative culture it provides privacy for the females and the young smokers. It could be that different smoker profiles require different cessation approaches with varying intensities future research could focus on that. However, more research is needed for this treatment modality as it is a relatively understudied behavioral intervention. Further research involving RCTs, which examines long-term quit rates and cost-effectiveness of telephone consultations, will be critical in establishing a telehealth platform to provide tobacco treatment services. As Qatar is witnessing an expansion in stop smoking treatment programs, our exploratory work will aid in contributing to the very limited tobacco cessation evidence base available in Qatar and act as a way forward to designing clinical trials for further formal assessments.

In the last years, factors influencing smoking cessation became the focus of many studies to improve the success of such treatments. In our regression analysis, the counseling method provided was an independent predictor of quitting with an adjusted odds ratio of 3.279 (95% CI: 1.191–9.026) for telephone counseling. In addition, attempt to quit was an independent predictor of quitting success regardless of the counseling method provided with an adjusted odds ratio of 4.724 (95% CI: 1.131–19.727). Self-reported cigarette consumption per day was a risk factor of quitting success with an adjusted odds ratio of 0.919 (95% CI: 0.874–0.966) in line with the findings of Bacha et al. [45]. Other studies have found similar results on the above-mentioned factors associated with quitting rate [45–47].

Our study has some limitations, given the number of subjects (small sample size) and demographics of smoking prevalence specific to this country (higher smoking prevalence among males) [4, 48]. The success rate was based on self-report with no biomarker assessment and may have been prone to recall bias. However, we only included and analyzed smokers who completed all follow-ups in both groups, whether by telephone or face-to-face. We also acknowledge the possible confounding bias with all the variables used in the model that would have affected the observed differences in quit rates between the two groups. Finally, a randomized control study design could have provided a more robust design for assessing the effectiveness of the two counseling methods. However, a retrospective study design was adopted due to limited resources. With these limitations, we believe this study makes a nascent contribution in exploring and assessing various smoking cessation treatment methods and for planning future studies in Qatar.

## Conclusion

In our study, smoking cessation using phone approach was reported to be effective, however our study comes with its limitations. Certainly, randomized controlled trials with due attention given to reduce bias are needed to determine the most effective method and further assessment needs to be conducted for more evaluation whether telephone counseling to be applied as stand-alone or in combination with the standard face-to-face care. Results obtained from this study highlight some factors which may be considered for successful quitting.

## Electronic supplementary material

Below is the link to the electronic supplementary material.

Supplementary Material 1

## Data Availability

Data availability upon reasonable request.
