# Supplementary material for: Comparison of quit rates among exclusive cigarette smokers at Tobacco Control Center in Qatar: telephone versus face-to-face consultation
Source: BMC Prim Care. 2025 May 15;26:168. doi: 10.1186/s12875-025-02858-2 (PMC12080017; doi:10.1186/s12875-025-02858-2)
Supplement: Supplementary file 1 — Supplementary Material 1 [file 12875_2025_2858_MOESM1_ESM.docx]

Supplementary Information

**Comparison of quit rates among exclusive cigarette smokers at Tobacco Control Center in Qatar: telephone versus face-to-face consultation**

Muslem M. Aljaafar,^a*^ Silva Kouyoumjian,^a^ Gafar Mahmoud,^a^ Ahmad AlMulla^a^

^a^ *Tobacco Control Center, WHO Collaborative Center, Department of Medicine, Hamad Medical Corporation, P.O. Box 3050, Doha, Qatar.*

*Muslem M. Aljaafar: [MMUSLEM@hamad.qa](mailto:MMUSLEM@hamad.qa)

Silva Kouyoumjian: [SKouyoumjian@hamad.qa](mailto:SKouyoumjian@hamad.qa)

Gafar Mahmoud: [GMahmoud1@hamad.qa](mailto:GMahmoud1@hamad.qa)

Ahmad AlMulla: [almulla@hamad.qa](mailto:almulla@hamad.qa)

*Corresponding author

1. **Methods**

**S1 Appendix**. The data collection tool in English
